# The spatiotemporal expression of multiple coho salmon ovarian connexin genes and their hormonal regulation *in vitro *during oogenesis

**DOI:** 10.1186/1477-7827-9-52

**Published:** 2011-04-19

**Authors:** Yoji Yamamoto, J Adam Luckenbach, Mollie A Middleton, Penny Swanson

**Affiliations:** 1 School of Aquatic & Fishery Sciences, University of Washington, Seattle, WA 98195, USA; 2 Northwest Fisheries Science Center, NOAA Fisheries, Seattle, WA 98112, USA

## Abstract

**Background:**

Throughout oogenesis, cell-cell communication via gap junctions (GJs) between oocytes and surrounding follicle cells (theca and granulosa cells), and/or amongst follicle cells is required for successful follicular development. To gain a fundamental understanding of ovarian GJs in teleosts, gene transcripts encoding GJ proteins, *connexins *(*cx*), were identified in the coho salmon, *Oncorhynchus kisutch*, ovary. The spatiotemporal expression of four ovarian *cx *transcripts was assessed, as well as their potential regulation by follicle-stimulating hormone (FSH), luteinizing hormone (LH) and insulin-like growth factor 1 (IGF1).

**Methods:**

Salmonid ovarian transcriptomes were mined for *cx *genes. Four gene transcripts designated *cx30.9*, *cx34.3*, *cx43.2*, and *cx44.9 *were identified. Changes in gene expression across major stages of oogenesis were determined with real-time, quantitative RT-PCR (qPCR) and *cx *transcripts were localized to specific ovary cell-types by in situ hybridization. Further, salmon ovarian follicles were cultured with various concentrations of FSH, LH and IGF1 and effects of each hormone on *cx *gene expression were determined by qPCR.

**Results:**

Transcripts for *cx30.9 *and *cx44.9 *were highly expressed at the perinucleolus (PN)-stage and decreased thereafter. In contrast, transcripts for *cx34.3 *and *cx43.2 *were low at the PN-stage and increased during later stages of oogenesis, peaking at the mid vitellogenic (VIT)-stage and maturing (MAT)-stage, respectively. In situ hybridization revealed that transcripts for *cx34.3 *were only detected in granulosa cells, but other *cx *transcripts were detected in both oocytes and follicle cells. Transcripts for *cx30.9 *and *cx44.9 *were down-regulated by FSH and IGF1 at the lipid droplet (LD)-stage, whereas transcripts for *cx34.3 *were up-regulated by FSH and IGF1 at the LD-stage, and LH and IGF1 at the late VIT-stage. Transcripts for *cx43.2 *were down-regulated by IGF1 at the late VIT-stage and showed no response to gonadotropins.

**Conclusion:**

Our findings demonstrate the presence and hormonal regulation of four different *cx *transcripts in the salmon ovary. Differences in the spatiotemporal expression profile and hormonal regulation of these *cx *transcripts likely relate to their different roles during ovarian follicle differentiation and development.

## Background

The growth and development of oocytes and surrounding follicle cells (i.e., granulosa and theca cells) occurs in a highly orchestrated and mutually dependent manner. Communication between these different ovarian cell-types is dependent on direct signaling mediated by gap junctions (GJs), in addition to signaling via endocrine and/or paracrine pathways [1,2]. Gap junctions are composed of an aggregate of intercellular membrane channels that allow the passage of molecules with a molecular weight of up to 1 kDa, including various second messengers such as cyclic AMP (cAMP) and inositol trisphosphate, and ions [3]. Each GJ channel is formed by two hemichannels (connexons), both of which are hexamers of connexin (Cx) protein subunits [4,5]. Two connexons from adjacent cells dock to form a GJ channel. In mammals, numerous studies indicate that ovarian GJ communication is involved in regulation of the meiotic arrest of oocytes, steroidogenesis, and apoptosis [6-9]. In fishes, by contrast, detailed information on the distribution, expression, and functions of ovarian GJs is largely unknown.

The few studies of ovarian GJs and *cx *gene transcripts in fishes were performed during late stages of maturation, prior to ovulation. Final maturation of the fish ovarian follicle involves a number of events including luteinizing hormone (LH)-dependent acquisition of oocyte maturational competence (OMC), LH induction of maturation-inducing hormone (MIH; typically either 17,20β-dihydroxy-4-pregnen-3-one or 17,20β,21-trihydroxy-4-pregnen-3-one) synthesis, and MIH-dependent meiotic resumption [10]. Previous studies with Atlantic croaker, *Micropogonias undulates*, and red seabream, *Pagrus major*, used electron microscopy to show that the number of ovarian GJs (between granulosa cells and oocytes, and amongst granulosa cells) increased during LH-dependent acquisition of OMC [11-13]. Increases in ovarian GJs were also induced by insulin-like growth factor 1 (IGF1) treatment in red seabream [12]. Further, Yamamoto et al. [14,15] found that culturing ovarian fragments with common GJ inhibitors prevented LH-induced acquisition of OMC in ayu, *Plecoglossus altivelis*, suggesting that ovarian GJ communication is essential for the LH-induced acquisition of OMC in this species. Thus, some ovarian GJs appear to be hormonally regulated and to have important roles during final maturation of the follicle in fishes. However, the function(s) and regulation of ovarian GJs during earlier stages of oogenesis, such as previtellogenic and vitellogenic stages, has not been studied.

So far, 21 human genes and 20 mouse *cx *genes have been identified [16]. In addition, 37 putative *cx *genes have been identified in the zebrafish genome [17]. Many *cx *genes show tissue- or cell-type-specific expression patterns and most organs express more than one *cx *[18]. According to Eastman's phylogenetic analysis, which was performed with the entire Cx family including human, mouse, and zebrafish Cx, *cx *genes can be classified into α, β, and γ groups, and potentially a fourth group containing human Cx62, mouse Cx57, and zebrafish Cx52.6 for example [17]. Studies in mammals have indicated that endocrine regulators of oogenesis such as follicle-stimulating hormone (FSH) and LH also regulate levels of *cx *gene transcripts in the ovary [9]. For example, up-regulation of *cx43 *transcripts in response to FSH was reported in a rat granulosa cell line [19], while LH had an inhibitory effect on the expression of *cx43 *in rat ovarian follicles *in vitro *[20]. Such gonadotropic regulation of *cx *gene transcripts has also been reported in teleosts. In red seabream, purified native FSH increased *cx32.3*, while LH increased *cx31.5 *and *cx32.3 *transcripts during acquisition of OMC [21,22]. In addition, human chorionic gonadotropin (hCG) elevated *cx32.2*, but not *cx32.7 *transcripts in Atlantic croaker during acquisition of OMC [23-25]. Thus, gonadotropins appear to regulate some ovarian *cx *gene transcripts during oocyte maturation in teleosts. Meanwhile, the regulation of ovarian *cx *gene transcripts by FSH, LH or IGF1 at earlier stages of oogenesis has not been examined.

The goals of this study were to identify and characterize ovarian *cx *gene transcripts in coho salmon, *Oncorhynchus kisutch*, determine whether levels of *cx *transcripts in the ovary change across stages of oogenesis, and to determine the subfollicular distribution of *cx *transcripts in the ovary. Finally, we determined whether FSH, LH or IGF1 regulate *cx *gene expression in previtellogenic and vitellogenic ovarian follicles. We used coho salmon as a model for this work because it is a semelparous species (spawns only once in its life and then dies) that exhibits synchronous follicle development. This unique reproductive trait allows for stage-specific analysis of a relatively homogenous clutch of ovarian follicles, which is not possible in iteroparous species. Furthermore, developmental profiles of FSH, LH, and IGF1 in the plasma are well characterized in salmon [26-29], giving biological relevance to any effects of these hormones on *cx *gene expression during a specific stage of ovarian development.

## Methods

### Animals and sampling

Coho salmon were reared at the Northwest Fisheries Science Center (Seattle, WA, USA) in 10-15°C recirculated fresh water and fed a standard ration (0.6-1.0% body weight/day) of a commercial diet (Skretting Feeds, Vancouver, BC, Canada). Lighting above the tanks was continually adjusted to match the natural photoperiod of Seattle (48°N). Stages of oogenesis were determined and confirmed by histological analyses using Campbell et al. [27] and Nagahama [30] as guides.

Perinucleolus (PN)-stage follicles were sampled from age-1+ salmon in August. Cortical alveolus (CA)-stage, lipid droplet (LD)-stage, early vitellogenic (VIT)-stage (with oocytes containing yolk granules), mid VIT-stage (with oocytes containing yolk globules), and preovulatory, maturing (MAT)-stage follicles were sampled from age-2+ salmon in March, June, July, August and December, respectively. The germinal vesicles of oocytes in the MAT-stage were migrating (just before germinal vesicle break down). Prior to tissue sampling, fish were euthanized in buffered tricaine methanesulfonate (0.05% MS-222, Argent Chemical, Redmond, WA) and body and ovary weight were recorded. A piece of ovary was collected for histological analysis, and other pieces were frozen in liquid nitrogen for RNA isolation and mRNA analyses. Fish used in the experiments were reared and handled according to the policies and guidelines of the University of Washington Institutional Animal Care and Use Committee (IACUC Protocol #2313-09).

### RNA isolation

For the across-stage comparisons of transcript levels, approximately 40-100 mg (wet wt.) pieces of ovarian tissue were homogenized in 1 ml Tri-Reagent/sample (Molecular Research Center, Cincinnati, OH) using a TissueLyser II (QIAGEN, Valencia, CA) and total RNA was isolated according to the manufacturer's instructions. Because of the large size of MAT-stage follicles (130-140 mg/follicle), 5 follicles/fish were homogenized in 7 ml of Tri-Reagent. For culture experiment 1 (see below), 40-70 mg of cultured ovarian tissue from each well was homogenized with 1 ml of Tri-Reagent. For culture experiment 2, one cultured follicle (late VIT-stage, 40-80 mg/follicle) from each well was homogenized in 1 ml of Tri-Reagent. Isolated total RNA samples were then diluted to ~250 ng RNA/ml in nuclease-free water.

Total RNA samples were then DNase treated using the Turbo DNA-Free kit's "rigorous" protocol (Applied Biosystems/Ambion, Austin, TX) where the amount of DNase enzyme and treatment time were doubled. RNA yields and quality were assessed by NanoDrop (ND-1000 Spectrophotometer; NanoDrop Technologies, Rockland, DE) and gel electrophoresis.

For the across-stage comparisons, mRNA was further isolated from total RNA samples to mitigate issues associated with comparing ovarian follicles during different stages of oogenesis, which may be dramatically different in size and RNA composition [31]. mRNA was isolated from 200 mg of total RNA/sample using the MicroPoly(A)Purist kit (Ambion, Austin, TX). As *in vitro *culture experiments were done with ovarian follicles of the same stage, total RNA was used for cDNA synthesis (see below).

### cDNA synthesis

For each sample, 500 ng of total RNA (for culture experiments) or 50 ng of mRNA (for across-stage comparisons of *cx *transcripts) was reverse transcribed in a 10-μl reaction with the Superscript II kit (Invitrogen, Carlsbad, CA). Other necessary components for reverse transcription (RT), such as random primers and RNase inhibitor, were purchased from Promega (Madison, WI). Negative control reactions were performed without the addition of the RT enzyme for a subset of the RNA samples.

### Identification of coho salmon connexins

To identify coho salmon ovarian *cx *gene transcripts, we conducted searches within our previous coho salmon ovarian cDNA libraries (Luckenbach et al. unpublished data) and located partial cDNAs for gene transcripts we later named *cx30.9*, *cx34.3*, and *cx44.9*. Partial cDNAs for *cx30.9 *and *cx44.9 *showed high homology to Atlantic salmon, *Salmo salar*, gap junction beta 6 protein (Gjb6, GenBank accession number; NM_001140280.1) and rainbow trout, *Oncorhynchus mykiss*, *cx *sequences (Accession numbers: TC152456 and TC137376) in the DFCI R. trout gene index database [32], respectively. Primers to amplify the complete coding sequence (CDS) of these two *cx *genes were designed within these salmonid fish sequences. The complete CDS for coho salmon *cx34.3 *was determined by constructing a contig from several coho salmon expressed sequence tags (ESTs) and then the entire sequence was confirmed by PCR. Although we did not find *cx43.2 *in our salmon ovarian cDNA libraries, rainbow trout *cx43 *(GenBank accession number DQ204869.1), which is a homologue of coho salmon *cx43.2*, had been previously studied in the trout ovary [33]. Thus, we cloned *cx43.2 *from the coho salmon ovary using primers designed from the trout cDNA sequence.

The primers for cloning each salmon *cx *are shown in Table 1. The RT-PCRs for cloning were carried out for 35 cycles as follows: 94°C for 30 s for denaturing, 52°C for 30 s for annealing and 72°C for 90 s for extension. The RT-PCR reactions consisted of 18 ng cDNA template (based on the mRNA loaded into the RT reactions), which was synthesized using mRNA of MAT-stage ovary. We used SuperTaq™ Polymerase (Ambion) according to the manufacturer's instructions. The resultant PCR products of expected size were inserted into a pGEM-T Easy vector (Promega, Madison, WI) or pCR^®^-XL-TOPO^® ^vector (Invitrogen), and completely sequenced on both DNA strands with an ABI PRISM 3100-Avant Genetic Analyzer.

**Table 1:** List of primers used for cloning and quantitative RT-PCR.

Step	Gene name	Strand	Sequence	Product size (bp)
cloning	*cx30.9*	Sense	5'-GAAATTCCGAGGAGGTGGTC-3'	1,088
		Antisense	5'-GCATGTTTGTCCAGGACTAC-3'	
cloning	*cx34.3*	Sense	5'-CTGAGAGACTGAGAGCTCCAAAC-3'	1,038
		Antisense	5'-AGACTGGCATGCGAATCATTG-3'	
cloning	*cx43.2*	Sense	5'-TCAAGAAGTGACGGAGAAAG-3'	1,278
		Antisense	5'-GTACTCTCTGTTCTCTGGCA-3'	
cloning	*cx44.9*	Sense	5'-AGCCTTAAAGAGCTGAGAGG-3'	1,273
		Antisense	5'-CGAGCAGGCATTAAGCAGGA-3'	
qPCR*	*cx30.9*	Antisense	5'-GGCTGGTGGAGTGTTTGTTC-3'	99
qPCR	*cx34.3*	Sense	5'-ACTACCTGTATGGCTTCACCCT-3'	284
		Antisense	5'-CTGGATCATCTGGTCTTTGTTC-3'	
qPCR	*cx43.2*	Sense	5'-ATGGCTGTTCCTCTCCCACT-3'	121
		Antisense	5'-CAGTTTTGCTCGTTGGCTTG-3'	
qPCR	*cx44.9*	Sense	5'-GTGACAGAAGGCTCTGTGAAG-3'	305
		Antisense	5'-ACACCGAGATGGAGAATCTC-3'	

*qPCR for *cx30.9 *was performed using *cx30.9 *cloning primer with the antisense qPCR primer for *cx30.9*.

### Quantitative PCR

For *cx *gene transcript analyses, we performed real-time quantitative RT-PCR (qPCR). The qPCR procedure was similar to that previously reported by Luckenbach et al [31,34]. The sequences of gene-specific primers (GSP) for qPCR are listed in Table 1. Reactions consisted of 1×Power SYBR Green PCR master mix (ABI), 150 nM of each gene-specific forward and reverse primer and 0.05 ng (based on the mRNA loaded into the RT reactions, for the across-stage comparisons of *cx *transcripts) or 0.5 ng (based on the total RNA loaded into the RT reactions, for culture experiment 1 and 2) of cDNA templates. Triplicate standard curve samples generated from a serial dilution of pooled mRNA from ovaries of six randomly selected previtellogenic coho salmon (for the across-stage comparisons of *cx *transcripts), or pooled total RNA from six randomly selected ovaries after being cultured 36 h with hormones (for culture experiment 1 and 2) were included in each plate. Results were analyzed using the relative standard curve method according to the manufacturer's instructions. Negative controls were included in each plate and consisted of either no RNA template (no template control, NTC) or RNA template that was not reverse transcribed (no amplification control, NAC). NACs reveal possible genomic DNA contamination in the RNA preparations. Negative control samples showed either no detectability or negligible values (>10 cycle separation from experimental samples). A melt curve analysis was included for each target gene to ensure that a single product was amplified, and in addition, a qPCR product was sequenced to verify that the target was successfully amplified.

Previous work in coho salmon showed that normalization of qPCR data to *elongation factor-1 alpha *(*ef1a*) generated results that best reflected transcript abundance on a per follicle basis in coho salmon follicle when comparing across different stages of oogenesis [31]. Therefore expression data for the *cx *transcripts were normalized to *ef1a *in this study. The levels of *ef1a *transcripts were stable (i.e., not significantly different) for the *in vitro *experiments and across four of the five stages (PN- to VIT-stage) of oogenesis included in the across-stage comparison (see Figure 2A inset). Due to the observed increase in *ef1a *at the MAT-stage, data for the *cx *genes were assessed both normalized to *ef1a *and un-normalized. Results overall for the two approaches were statistically very similar and therefore *ef1a *normalized data are shown for the *cx *transcripts. The primers for *ef1a *were the same as previously reported [31].

### In situ hybridization

Transcripts for all four *cx *gene transcripts were localized by in situ hybridization (ISH) in PN-, CA-, LD-, early VIT- and MAT-stage ovarian follicles. However, ISH was technically difficult to perform with MAT-stage follicles due to the large amount of yolk present in the oocytes, and results are therefore not shown for this stage.

The cDNA fragments containing the entire CDS -- that is, nucleotides 1- 1,088, 1-1,038, 1-1,278, and 1-1,273 for *cx30.9*, *cx34.3*, *cx43.2*, and *cx44.9*, respectively -- were used as templates for the synthesis of RNA probes. Sense and antisense RNA probes for each *cx *were transcribed *in vitro *using digoxigenin-labeled UTP (Roche Diagnostics, Basel, Switzerland) and T7 RNA polymerase (New England Biolabs, Ipswich, MA). To prepare tissues for ISH, ovarian samples were fragmented into several pieces and fixed in 4% paraformaldehyde (Electron Microscopy Sciences, Hatfield, PA) with gentle shaking at room temperature for 16-36 h. Fixed ovaries were rinsed three times with phosphate buffered saline (PBS) over 45 min, sequentially dehydrated through a methanol series (25%, 50%, and 75% methanol/PBS), and stored in 100% methanol at - 20°C until use.

A portion of each sample was embedded in paraffin wax and cut into 5-μm serial sections using a microtome. Paraffin sections were mounted on SUPERFROST^® ^Plus microscope slides (Thermo Scientific, Portsmouth, NH), dewaxed, and dehydrated by immersion in a xylene-ethanol series. Slides were either stained with hematoxylin and eosin (VECTOR LABORATORIES, Burlingame, CA) or processed for ISH with DIG-labeled RNA probes. Sections for ISH were permeabilized, post-fixed with 4% paraformaldehyde at room temperature for 20 min, and treated with 5 mg/ml proteinase K at 37°C for 10 min. The sections were subsequently acetylated, and then incubated with a hybridization mixture of 0.0125-0.2 mg/ml RNA probe, 50% formamide, 2× saline-sodium citrate (SSC) (pH 4.5), 50 mg/ml transfer RNA (tRNA), 50 mg/ml heparin, 1% sodium dodecyl sulfate (SDS), and 10% dextran sulfate. After hybridization at 65°C for 16 h, the sections were washed as follows: twice in 5× SSC/50% formamide at 65°C for 30 min, three times in 2× SSC/50% formamide at 65°C for 30 min, and once in 1× SSC/25% formamide:1× Tris buffered saline containing 0.1% Tween-20 (TBST) at room temperature for 30 min. Unbound probes were digested using 20 mg/ml RNase A to reduce background signals. After RNase digestion at 37°C for 30 min, the sections were placed in NTE buffer (500 mM NaCl, 10 mM Tris-HCl pH 8.0, 1 mM ethylenediamine tetraacetic acid) at 37°C for 5 min before being washed three times in 0.5× SSC at 65°C for 20 min, three times in 1× TBST at room temperature for 5 min, and in blocking solution (1×TBST and 2% Blocking Reagent; Roche Diagnostics) at room temperature for 1 h. Subsequently, the sections were incubated with the Fab fragment of an anti-DIG-alkaline phosphatase-conjugated antibody (Roche Diagnostics) diluted 1:2000 with blocking solution at 8°C for 16 h. Finally, each section was rinsed three times in TBST containing 1 mM Levamisole (Sigma-Aldrich, St. Louis, MO) for 5 min. The sections were then incubated in a NTMT solution (100 mM NaCl, 100 mM Tris-HCl pH 9.5, 50 mM MgCl_2_, 0.1% Tween 20, 1 mM Levamisole) containing 0.0035% nitroblue tetrazolium (NBT; Roche Diagnostics) and 0.0018% 5-bromo-4-chloro-3-indolyl phosphate (BCIP; Roche Diagnostics) at room temperature in the dark. After the color reaction had occurred, the slides were sealed with CYTOSEAL™ XYL (Thermo Scientific).

### Ovarian cultures

Culture experiments were conducted as described previously [34,35] to assess the effects of various hormones on ovarian *cx *gene expression. Animals were anaesthetized as above and the ovaries were removed, weighed, and held in chilled Leibovitz L-15 medium (Invitrogen, Carlsbad, CA) prior to dissection of follicles. Highly-purified coho salmon FSH and LH used in the experiments were obtained according to Swanson et al. [36]. Human recombinant IGF1 was purchased from Bachem (Torrance, CA). All hormones were solubilized in 20 mM phosphate buffered saline supplemented with 0.2% bovine serum albumin (BSA, RIA grade; Sigma-Aldrich) and then dissolved directly in the culture medium. Ovarian tissue fragments from a fish were distributed into 24-well polystyrene culture plates so that each treatment received one tissue fragment from each of the fish. Each treatment therefore included ovarian follicles from 6 different fish. Culture wells contained 1 ml of L-15 medium supplemented with 0.2% BSA and tissues were pre-incubated at 14°C for 2 h with gentle orbital shaking at 100 rpm. After the pre-incubation, the medium was removed and replaced with fresh L-15 medium containing either no hormone or hormone as described below. Time 0 h ovaries (initial) were collected and snap frozen in liquid nitrogen just after the pre-incubation for later RNA isolation.

#### Culture experiment 1; FSH and IGF1 effects on ovarian cx genes

This experiment was conducted in mid-June 2010 with LD-stage ovaries, because previous studies with coho salmon showed that levels of plasma FSH begin to increase in early spring (generally, Feb-May) and reach peak levels in late summer (Aug-Sept) [26]. The mean fish body weight (± SEM) was 595.9 ± 29.0 g, fork length was 36.2 ± 0.7 cm, paired ovary weight was 6.42 ± 0.62 g, and gonadosomatic index (GSI; gonad weight/body weight*100) was 1.07 ± 0.06%. Approximately 40-70 mg of ovarian tissue/well was incubated with or without hormones. FSH concentrations were 0 (control), 10, 50, 100, or 500 ng/ml and IGF1 concentrations were 0 (control), 1, 10, or 100 nM. Cultures were maintained for 36 h based on results of a previous time course study [34], which demonstrated that several ovarian genes affected by FSH showed a difference from controls at this time point. After the experiment, ovarian tissues were dabbed on lens paper to remove excess liquid, weighed, and snap frozen in liquid nitrogen for later RNA isolation.

#### Culture experiment 2; LH and IGF1 effects on ovarian cx genes

This experiment was conducted in early October 2010 with late VIT-stage ovaries, because previous studies with coho salmon showed that plasma LH levels begin to increase slightly in fall (generally Oct/Nov in our hatchery), prior to the ovulatory surge [26]. The mean fish body weight (± SEM) was 1152.8 ± 90.9 g, fork length was 44.0 ± 0.6 cm, paired ovary weight was 110.3 ± 20.3 g, and GSI was 9.1 ± 1.1%. Because of the large size of late VIT-stage follicles (40-80 mg/follicle), three follicles/well were cultured with or without hormones for 36 h. The LH concentrations were 0 (control), 10, 50, 100, or 500 ng/ml and IGF1 concentrations were the same as in experiment 1.

### Measurement of medium E2 levels

In salmon, both gonadotropins have been shown to stimulate production of estradiol-17β (E2) by ovarian follicles *in vitro *[34,37] and E2 had a biphasic effect on transcripts for ovarian *cx *genes in Atlantic croaker [25]. Furthermore, IGF1 can modulate aromatase activity [38]. Thus, it is informative to know how these hormones affected ovarian E2 production, which in turn may have influenced *cx *gene expression. After the 36-h cultures, the medium from each well was collected and stored at -80°C until later E2 measurement. Samples were heat treated at 80°C for 1 h, centrifuged at 15,700 × g for 7 min, and supernatants were transferred to a fresh tube [34]. Medium E2 levels were then determined by radioimmunoassay (RIA) as previously described [39].

### Statistical analysis

The across-stage *cx *gene expression data and *in vitro *ovarian incubation data were subjected to one-way analysis of variance followed by Tukey multiple mean comparison tests. Data were log_10 _transformed when necessary to meet normality and equal variance assumptions and reported as means ± SEM. Results for initial and control samples from the ovarian incubation experiments were compared by unpaired t-tests. All statistical analyses were conducted using the SPSS 11.0 microcomputer software package (SPSS, Chicago, IL).

## Results

### Isolation and characterization of coho salmon cx cDNAs

**Figure 1:**
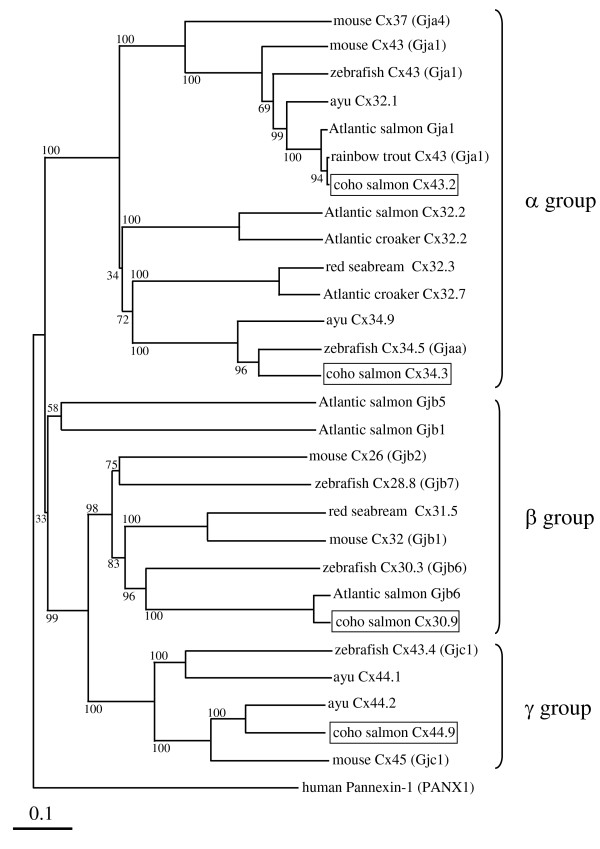
**Phylogenetic relationship between coho salmon and other known Cx proteins**. A neighbor-joining (NJ) distance tree comparing full-length amino acid sequences is shown. Bootstrap values (%) are shown for the NJ analyses. The GenBank accession numbers of the aligned deduced amino acid sequences of Cx proteins are as follows: Atlantic croaker Cx32.2 (L31542.1) and Cx32.7 (L31541.1), Atlantic salmon Cx32.2 (BT049836.1), gap junction protein alpha 1; Gja1 (BT059188.1), gap junction protein beta 1; Gjb1 (BT044946.1), gap junction protein beta 5; Gjb5 (BT057893.1) and gap junction protein beta 6; Gjb6 (NM_001140280.1), ayu Cx32.1 (DQ487733.1), Cx34.9 (DQ487730.1), Cx44.1 (DQ487731.1) and Cx44.2 (DQ487732.1), mouse, *Mus musculus*, Cx26 (also known as gap junction protein beta 2; Gjb2, AAA37276.1), Cx32 (also known as gap junction protein beta 1; Gjb1, AAA37296.2), Cx37 (also known as gap junction protein alpha 4; Gja4, NP_032146.1), Cx43 (also known as gap junction protein alpha 1; Gja1, NM_010288.3) and Cx45 (also known as gap junction protein gamma 1; Gjc1, NM_008122.3), rainbow trout Cx43 (also known as gap junction protein alpha 1; Gja1, DQ204869.1), red seabream Cx31.5 (AB037933.1) and Cx32.3 (AB048251.1), and zebrafish Cx28.8 (also known as gap junction protein beta 7; Gjb7, NP_001020715.1), Cx30.3 (also known as gap junction protein beta 6; Gjb6, NP_997990.2), Cx34.5 (also known as gap junction protein alpha a; Gjaa, NP_001025371.1), Cx43 (also known as gap junction protein alpha 1; Gja1, NP_571113.1), and Cx43.4 (also known as gap junction protein gamma 1; Gjc1, AAB36619.1). Human, *Homo sapiens*, Pannexin-1 (PANX1; NP_056183.2) was used as the outgroup.

cDNAs encoding 4 salmon *cx *genes were obtained with GSPs (Table 1). The *cx30.9 *cDNA (GenBank Accession No. HQ315553) was 1,088 bp and 272 aa, *cx34.3 *(HQ315554) was 1,038 bp and 298 aa, *cx43.2 *(HQ315555) was 1,278 bp and 383 aa, and *cx44.9 *(HQ315556) was 1,273 bp and 399 aa. From the predicted Cx amino acids sequences, the expected molecular weights of the proteins would be 30.9, 34.3, 43.2, and 44.9 kDa. Therefore, following the nomenclature system proposed by Beyer et al. [40], we named the genes accordingly. The homologies of amino acid sequences among the coho salmon *cx *genes were less than 55%. NCBI protein BLAST [41] searches revealed that coho salmon *cx30.9*, *cx34.3*, *cx43.2 *and *cx44.9 *sequences had the highest homology with Atlantic salmon Gjb6 (94.5%), zebrafish, *Danio rerio*, Cx34.5 (76.5%; GenBank accession number; NP_001025371.1), rainbow trout Cx43 (99.7%), and ayu Cx44.2 (72.5%; GenBank accession number; DQ487732.1), respectively. The amino acid sequences obtained for the four *cx *gene transcripts included all the characteristic features of Cx family proteins, including two *cx *consensus sequences, C(D, N)TXQPGCX_2_VCYD and CX_3or4_PCX_3_(L, I, V, M)(D, E, N)C(F, Y)(L, I, V, M)(S, A)(K, R)P, in the predicted extracellular loops (data not shown). Hydropathy analysis revealed that the coho salmon Cx proteins encoded by these genes would contain four hydrophilic domains and five hydrophobic domains that are typical of known Cx proteins (data not shown). Furthermore, phylogenetic analysis, was performed by the neighbor-joining method with ClustalX multiple alignment algorithm using NJPLOT software [42,43]. This revealed that Cx34.3 and Cx43.2 are α-type, Cx30.9 is a β-type, and Cx44.9 is a γ- type *cx *(Figure 1). The zebrafish and mouse Cxs in our analysis were classified in the same groups as previously reported [17].

### Ovarian cx transcript levels during oogenesis

**Figure 2:**
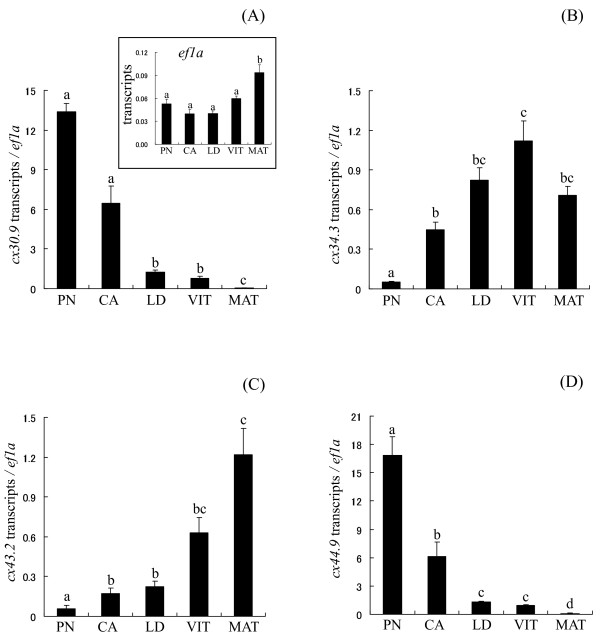
**Temporal expression patterns of four *cx *mRNAs during oogenesis**. The expression levels of *cx30.9 *(A), *cx34.3 *(B), *cx43.2 *(C), and *cx44.9 *(D) were normalized to *ef1a *(inset of panel A). PN, perinucleolus stage follicles; CA, cortical alveolus stage follicles; LD, lipid droplet stage follicles; VIT, mid-vitellogenic stage follicles; MAT, preovulatory, maturing stage follicles. Bars not sharing the same letter are significantly different (*P *< 0.05; n = 4/group of PN-, CA-, LD-, and VIT-stages, n = 3/group of MAT-stage, mean ± SEM).

The levels of transcripts for *cx30.9 *and *cx44.9 *showed a similar profile across-stages of oogenesis where levels were highest at the PN-stage and steadily declined thereafter (Figure 2A and 2D). Levels of transcripts for *cx34.3 *were lowest at the PN-stage, increased leading up to vitellogenesis, and reached peak levels by the mid VIT-stage (Figure 2B). Levels of *cx43.2 *transcripts remained low during previtellogenic stages, increased during vitellogenesis and peaked by the MAT-stage (Figure 2C).

### Intraovarian distribution of cx mRNAs

**Figure 3:**
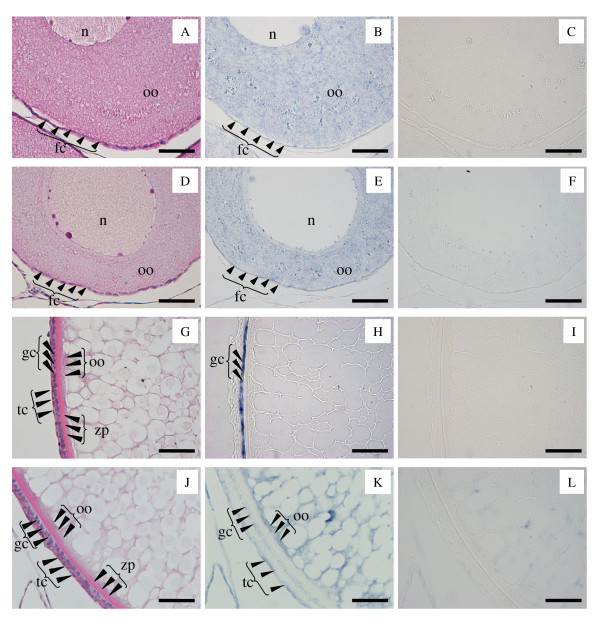
**Subfolliclular distribution of mRNAs for *cx30.9*, *cx44.9*, *cx34.3*, and *cx43.2 *in coho salmon ovaries**. Perinucleolus (PN)-stage follicles were used for *cx30.9 *and *cx44.9 *analyses. Lipid droplet (LD)-stage follicles were used for *cx34.3 *and *cx43.2 *analyses. Sequential sections were stained with hematoxylin and eosin (*cx30.9*, A; *cx44.9*, D; *cx34.3*, G; and *cx43.2*, J), and hybridized with antisense (*cx30.9*, B; *cx44.9*, E; *cx34.3*, H; and *cx43.2*, K) and sense (*cx30.9*; C, *cx44.9*; F, *cx34.3*; I, and *cx43.2*; L) probes. n, nuclear; oo, ooplasm; fc, follicle cells; tc, theca cells; gc, granulosa cells; zp, zona pellucida. Scale bars = 50 μm.

The results of ISH for each *cx *are shown in Table 2 and Figure 3. ISH using PN-stage ovaries indicated that *cx30.9 *(Table 2 and Figure 3B) and *cx44.9 *(Table 2 and Figure 3E) transcripts were present in oocytes and follicle cells. As follicle cell layers at the PN-stage were very thin, it was not possible to distinguish whether both the theca and granulosa cells expressed these *cx *transcripts (Figure 3B and 3E). The signals for *cx30.9 *and *cx44.9 *transcripts were also detected in oocytes from the CA- to VIT-stage, however the signals in follicle cells were not detected in the CA-stage or thereafter (Table 2). ISH indicated that *cx34.3 *mRNA was only expressed in the follicle cells (only granulosa cells through the CA- to the VIT-stage, Table 2 and Figure 3H). Transcripts for *cx43.4 *were localized to follicle cells (both theca and granulosa cells at the CA-, LD- and early VIT-stages) and within oocytes (Table 2 and Figure 3K).

**Table 2:** Spatial expression profiles of *cx *transcripts in the coho salmon ovary

*cx*	Localization	PN-stage	CA-stage	LD-stage	VIT-stage
*cx30.9*	oocyte	+	+	w	w
	follicle cells	+	G: x, T: x	G: x, T: x	G: x, T: x
*cx34.3*	oocyte	x	x	x	x
	follicle cells	+	G: +, T: x	G: +, T: x	G: +, T: x
*cx43.2*	oocyte	+	+	+	+
	follicle cells	+	G: +, T: +	G: +, T: +	G: +, T: +
*cx44.9*	oocyte	+	+	w	w
	follicle cells	+	G: x, T: x	G: x, T: x	G: x, T: x

Each mark indicates signal levels of *cxs *by ISH. +, signal; w, weak signal; x, signal undetectable. PN, perinucleolus stage follicles; CA, cortical alveoli stage follicles; LD, lipid droplet stage follicles; VIT, early-vitellogenic stage follicles. G, granulosa cells; T, theca cells.

### Culture experiment 1: Effects of FSH and IGF1 on ovarian cx gene expression

**Figure 4:**
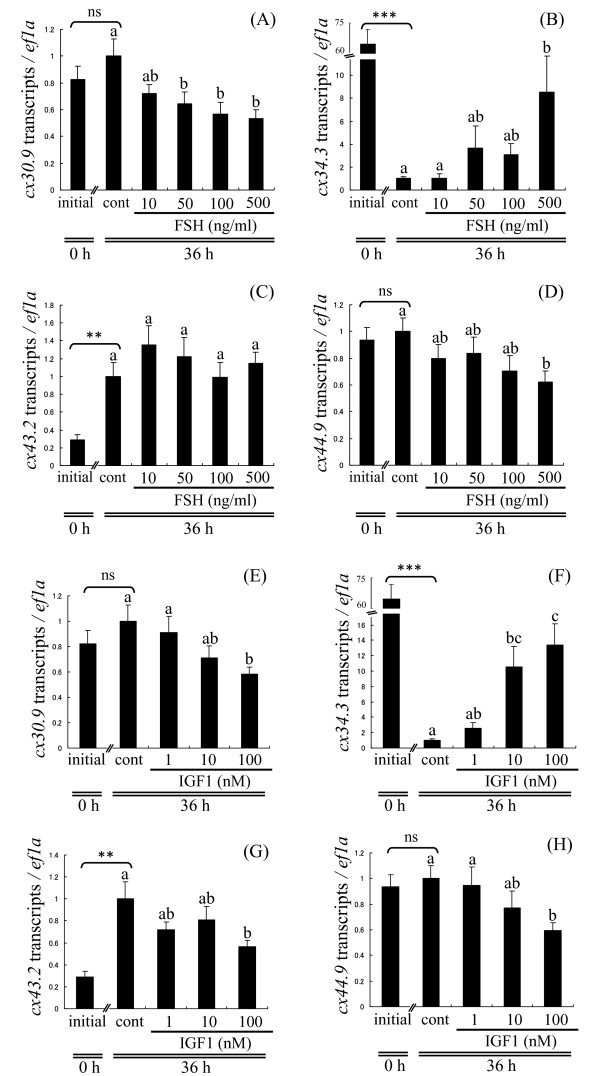
**Hormonal regulation of ovarian *cx *genes *in vitro*: culture experiment 1**. Effects of varying FSH (10, 50, 100, or 500 ng/ml) and IGF1 (1, 10, or 100 nM) concentrations on levels of transcripts for *cx30.9 *(A, E), *cx34.3 *(B, F), *cx43.2 *(C, G), *cx44.9 *(D, H) in lipid droplet (LD)-stage follicles. Data are expressed as the mean ± SEM (n = 6) and presented relative to the expression of controls (cont; Time 36 h, no hormones, set to 1). Bars not sharing the same letter are significantly different (P < 0.05). Asterisks denote values that significantly differ between initial (Time 0, no hormones) and control (cont; Time 36 h, no hormones), **P < 0.01, ***P < 0.001. ns, no significant difference.

In LD-stage follicles, FSH significantly reduced transcript levels for *cx30.9 *and *cx44.9 *in a concentration-dependent manner (Figure 4A and 4D). In contrast, transcripts for *cx34.3 *increased in a concentration-dependent manner, reaching a more than 8-fold maximum elevation relative to control when treated with 500 ng FSH/ml (Figure 4B). FSH did not affect *cx43.2 *levels at any concentration (Figure 4C). IGF1 had effects similar to FSH on ovarian *cx *expression. Transcripts for *cx30.9 *and *cx44.9 *were suppressed in a concentration-dependent manner, but were only significantly down-regulated relative to controls at the highest concentration, 100 nM IGF1 (Figure 4E and 4H). Transcripts for *cx43.2 *were significantly suppressed by 100 nM IGF1 relative to controls (Figure 4G). In contrast, IGF1 elevated transcripts for *cx34.3 *in a concentration-dependent manner reaching a more than 13-fold maximum increase relative to control when treated with 100 nM IGF1 (Figure 4F).

Levels of *cx *transcripts in LD-stage ovaries cultured in control medium for 0 h (initial) and 36 h showed different patterns. Transcripts for *cx30.9 *(Figure 4A) and *cx44.9 *(Figure 4D) did not change significantly between initial and control. Transcripts for *cx34.3 *decreased dramatically reaching a more than 64-fold maximum decline by 36 h (Figure 4B). In contrast, transcripts for *cx43.2 *increased more than 3-fold relative to initial levels after 36 h in culture (Figure 4C).

### Culture experiment 2: Effects of LH and IGF1 on ovarian cx gene expression

**Figure 5:**
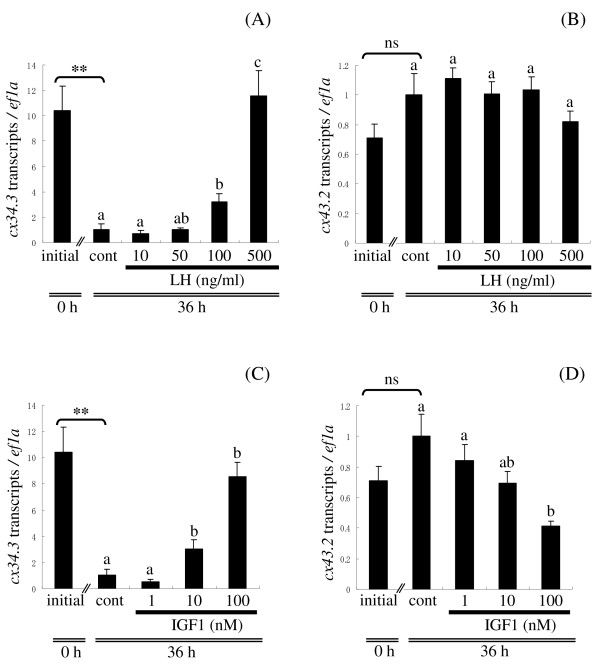
**Hormonal regulation of ovarian *cx *genes *in vitro*: culture experiment 2**. Effects of varying LH (10, 50, 100, or 500 ng/ml) and IGF1 (1, 10, or 100 nM) concentrations on transcripts for *cx34.3 *(A, C) and *cx43.2 *(B, D) at the late vitellogenic (VIT)-stage. Data are expressed as the mean ± SEM (n = 6) and presented relative to the expression of controls (cont; Time 36 h, no hormones, set to 1). Bars not sharing the same letter are significantly different (P < 0.05). Asterisks denote values that significantly differ between initial (Time 0, no hormones) and control samples, **P < 0.01. ns, no significant difference.

In late VIT-stage follicles, LH increased the level of transcripts for *cx34.3 *in a concentration-dependent manner, reaching a more than 11-fold increase relative to control when treated with 500 ng LH/ml (Figure 5A). In contrast, LH did not affect transcripts for *cx43.2 *at any concentration tested (Figure 5B). IGF1 elevated transcripts for *cx34.3 *in a concentration-dependent manner reaching a more than 8-fold maximum increase relative to control when treated with 100 nM IGF1 (Figure 5C). In contrast, IGF1 suppressed transcripts for *cx43.2 *in a concentration-dependent manner, but this was only significant with 100 nM IGF1 (Figure 5D). Transcripts for *cx30.9 *and *cx44.9 *were very low at the late-VIT stage, and neither LH nor IGF1 altered levels of these transcripts at any concentration tested (data not shown).

Levels of transcripts for each *cx *gene in late VIT-stage ovaries cultured in control medium for 0 h (initial) and 36 h showed different patterns. Notably, transcripts for *cx34.3 *decreased more than 10-fold after 36 h in culture (Figure 5A or 5C), while transcripts for *cx43.2 *increased after the 36-h incubation, but this was not significant (Figure 5B or 5D). Transcripts for *cx30.9 *and *cx44.9 *were very low in the initial sample and did not change after 36 h culture (data not shown).

### In vitro ovarian E2 production

**Figure 6:**
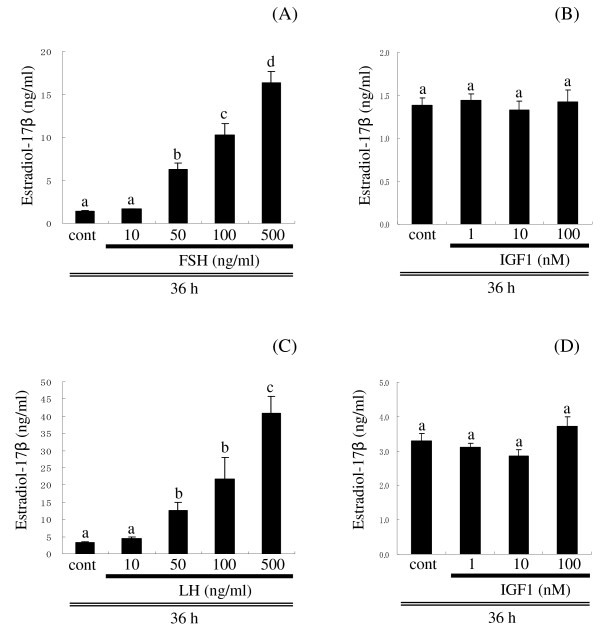
***In vitro *ovarian E2 production**. Effect of varying FSH (A) and IGF1 (B) concentrations on in vitro E2 production by lipid droplet (LD)-stage follicles (culture experiment 1) and varying LH (C) and IGF1 (D) concentrations on in vitro E2 production by late vitellogenic (VIT)-stage follicles (culture experiment 2). Incubations were for 36 h. Data are expressed as the mean ± SEM (n = 6). Bars not sharing the same letter are significantly different (P < 0.05).

Medium E2 levels increased in a concentration-dependent manner in response to FSH (culture experiment 1, Figure 6A) and LH (culture experiment 2, Figure 6C). In contrast, IGF1 had no effect on E2 production at any concentration in both experiments (Figure 6B and 6D).

## Discussion

In this study, we have shown that multiple *cx *gene transcripts, designated *cx30.9*, *cx34.3*, *cx43.2 *and *cx44.9*, are expressed in coho salmon ovaries, have distinct developmental expression patterns, and differ in their hormonal regulation. The sequence homologies, consensus sequences, and predicted topologies of the four deduced proteins are in strong agreement with the predicted structural traits of Cx family proteins [4]. Such multiple expression of *cx *gene transcripts in the ovary is well known in mammals [9]; however, interrelationships between various *cx *isoforms to form a connexon and ultimately GJs are poorly understood. GJs are composed of two connexons, both of which are hexamers of Cx proteins. As most cell types express more than one *cx *isoform, a connexon could potentially be composed of either one type of Cx (homomeric) or more than one type of Cx (heteromeric) [44]. When two identical connexons dock, they form a homotypic GJ, while when two different connexons dock, they form a heterotypic GJ [44]. We did not examine the functional aspects of the four different Cx proteins in coho salmon; however, previous studies in two Perciform fishes suggest that heterotypic GJs have much lower functional activity than homotypic GJs [45,46]. To determine the compatibility between four different Cx proteins in coho salmon, further analyses using an *in vitro *cell expression system and dye transfer assay [47,48] will be needed.

Although our phylogenetic analysis showed that *cx30.9 *and *cx44.9 *were classified in the β group and γ group, respectively, the results of spatiotemporal analyses and hormone effects on these genes were similar. Transcripts for *cx30.9 *and *cx44.9 *were highly expressed at the PN-stage, however these transcripts decreased dramatically by the LD-stage and remained low thereafter. Further, these genes were expressed in follicle cells (theca and/or granulosa cells) and oocytes only at the PN-stage. These data suggest that GJs composed of *cx30.9 *and *cx44.9 *could have important roles during early oogenesis and could form GJs between follicle cells and the oocyte, and amongst follicle cells at the PN-stage. To date, there are no *cx *isoforms identified in fishes that show the exact spatiotemporal expression pattern as coho salmon *cx30.9 *and *cx44.9*. However, in ayu, two *cx *isoforms designated *cx44.1 *and *cx44.2*, that have the highest homology to coho salmon *cx44.9*, were similarly expressed at the highest levels during early oogenesis [49], but were only expressed in the oocyte and levels did not decline at more advanced stages of oogenesis as seen in salmon. Although coho salmon *cx30.9 *and *cx44.9 *share some similarities to ayu *cx44.1 *and *cx44.2*, the difference in localization of the *cx *transcripts and the low levels of these two coho salmon *cx *transcripts during the LD-through MAT- stage suggest they are unlikely to have the same function as the ayu *cx *genes.

The gene encoding *cx34.3 *was classified in the α group in our phylogenetic analysis, began to increase at the CA-stage, reached maximum levels at the mid VIT-stage and remained high thereafter. Further, ISH revealed that *cx34.3 *was localized only in granulosa cells. These data suggest that *cx34.3 *may compose homotypic GJs, only between granulosa cells, which could have important roles in vitellogenesis and final maturation. In teleosts, it is generally accepted that granulosa cells produce steroid hormones such as E2 and MIH from their respective precursors [50]. In mammals, it is well known that the hormone-producing cells are also connected by functional GJs and are needed for the cells to biosynthesize, store and release hormone effectively [51,52]. Gap junctional communication amongst granulosa cells may have an important role in steroidogenesis [12,53]. Thus, in coho salmon, GJs composed of *cx34.3 *between granulosa cells may have an important role in ovarian steroidogenesis, but further research will be needed to establish this.

In contrast to *cx34.3*, transcripts for *cx43.2 *increased later in vitellogenesis, reached maximum levels in the preovulatory follicles, and were localized in both follicle cells (theca and granulosa cells at CA-, LD- and early VIT-stages) and oocytes. These data suggest that *cx43.2 *could compose homotypic GJs, between the follicle cells and the oocyte, and between the follicle cells, and that GJs formed by *cx43.2 *may be involved in late vitellogenesis and final maturation. The observed increase in *cx43.2 *at the MAT-stage was consistent with a previous report of rainbow trout *cx43 *[33], a homologue of coho salmon *cx43.2*. Phylogenetic analysis revealed that coho salmon *cx43.2 *and trout *cx43 *were both classified in the α group. The temporal expression pattern and follicular localization of coho salmon *cx43.2 *showed a similar pattern to that of ayu *cx34.9 *[49]. In ayu, *cx34.9 *appears to contribute to formation of GJs between the oocyte and the granulosa cells, and may have an important role in transmitting the LH-signal received in the follicle cells to the oocyte via a second messenger such as cAMP during acquisition of OMC [14,15,49]. Although we have no empirical data on the function of coho salmon *cx43.2*, it may have a similar role to ayu *cx34.9 *given the similarity of their spatiotemporal expression patterns.

Hormonal regulation of *cx *gene transcripts has been reported in several fishes. However, previous studies of the hormonal regulation of ovarian *cx *gene transcripts mainly focused on MAT-stage follicles [21-25]. To assess hormonal regulation of *cx *gene transcripts during various stages of oogenesis, we performed two ovarian culture experiments using previtellogenic (LD-stage; culture experiment 1) and late VIT-stage (culture experiment 2) follicles. At the LD-stage, both FSH and IGF1 increased transcript levels for *cx34.3*, but decreased transcripts for *cx30.9 *and *cx44.9*. In late VIT-stage follicles, LH and IGF1 had a stimulatory effect on transcripts for *cx34.3*. While LH had no effect on levels of transcripts for *cx43.2*, IGF1 showed a clear inhibitory effect. These data suggest that gonadotropins and IGF1 regulate ovarian *cx *trancripts in a *cx- *and stage-specific manner, and may influence GJ formation and therefore communication within the ovary. Although the stimulatory effect of IGF1 on the number of GJs has been reported in red seabream ovary [12], to our knowledge this is the first report of IGF1 regulation of ovarian *cx *gene expression. As IGF1 receptors were found in granulosa cells of coho salmon [38], it is possible that IGF1 (either endocrine or paracrine IGF) regulates *cx34.3 *gene expression in granulosa cells. The up- and down- regulation of specific *cx *genes by gonadotropins shown in the present study is consistent with previous studies in Atlantic croaker [23-25]. Although the mechanisms of ovarian *cx *activation by hormones were not addressed in the present study, studies of Atlantic croaker revealed that gonadotopic regulation of *cx *genes was mediated by the cAMP-protein kinase A transduction pathway [24,54]. Obviously, further promoter studies are needed to elucidate the role of second messenger systems or other transcription factors in the regulation of *cx *gene expression by gonadotropins and IGF1 in the ovary of coho salmon.

Our results show that both FSH and LH, but not IGF1, stimulated *in vitro *production of ovarian E2, thus we cannot rule out the possibility that the observed effects of gonadotropins were mediated by steroids. In mammals, many studies indicated that steroid hormones regulate *cx *gene expression [55-57]. For example, in the ovariectomized rat endometrium, a high amount of progesterone in combination with low E2 levels suppressed transcripts for *cx26 *and *cx43*, but higher E2 levels had no effect on *cx26 *expression [57]. In Atlantic croaker, E2 had a biphasic effect on *cx32.7 *[25]. At a low concentration, E2 had no effect on *cx32.7 *transcripts, but at high concentrations, it inhibited expression [25]. Thus, E2 appears to regulate *cx *gene expression in teleosts as well. To clarify the involvement of steroid hormones in the regulation of ovarian *cx *gene expression, further *in vitro *culture experiments using inhibitors of E2 synthesis or other steroid hormones such as progesterone and testosterone will be needed.

The developmental patterns and hormonal regulation of *cx *gene expression were consistent with what is known about plasma levels of FSH, LH, and IGF1 during the reproductive cycle of salmon. For example, transcripts for *cx34.3 *began to increase at the CA- to LD-stage and peaked during mid vitellogenesis. This expression profile is consistent with plasma FSH [26,27] and IGF1 profiles in female coho salmon [27,29] and our results indicate that both of these hormones stimulate ovarian *cx34.3 *expression *in vitro*. Plasma levels of FSH [26] and IGF1 [28] in salmon decrease at final oocyte maturation, while plasma LH levels increase during this period [26]. Our results indicate that at this stage, LH increased expression of *cx34.3*. Taken together, high expression of *cx34.3 *at the LD - to VIT-stage could be regulated by FSH and IGF1, and then at the MAT-stage, LH could maintain high expression of *cx34.3*. Interestingly, incubation of LD-stage ovarian follicles in control medium without any hormones for 36 h reduced transcripts for *cx34.3 *more than 64-fold relative to the initial levels, and this reduction did not fully recover by incubation with FSH and IGF1, even at the highest hormone concentrations. These data suggest that *cx34.3 *is regulated by a number of factors *in vivo*, including FSH and IGF1, and perhaps other unidentified factors at this stage. Since we did not test any combination of hormones in our experiments, we do not yet know if there are additive or synergistic effects of gonadotropins and IGF1.

In contrast to *cx34.3*, FSH and LH did not have any effect on levels of *cx43.2 *transcripts. It was somewhat surprising that LH, in particular, did not show any effect on this gene, despite the expression profile of *cx43.2 *being similar to that of plasma LH [26]. Although the existence of other regulators of *cx43.2 *remains a possibility, one potential scenario to explain ovarian *cx43.2 *regulation in coho salmon is inhibitory regulation by IGF1. Both culture experiments revealed that only IGF1 inhibited *cx43.2 *expression. In salmon, plasma IGF1 levels typically increase during early secondary oocyte growth and decrease at the preovulatory stage [27-29]. Temporal expression analyses revealed that transcripts for *cx43.2 *were relatively low until the mid VIT-stage, but significantly increased by the MAT-stage. Thus, IGF1 may inhibit *cx43.2 *expression through most of oogenesis, and the natural decrease in plasma IGF1 prior to final oocyte maturation may induce elevations in *cx43.2 *expression. Notably, incubation of ovarian follicles in control medium for 36 h induced more than a 3-fold increase in this gene relative to initial samples in culture experiment 1. These *in vitro *data as well as the developmental pattern *in vivo *support the idea that *in vivo *factors negatively regulate *cx43.2 *expression, and that one of these factors may be IGF1.

At the LD-stage, transcripts for *cx30.9 *and *cx44.9 *were down-regulated by both FSH and IGF1 *in vitro*. Temporal expression analyses of these transcripts revealed that their expression decreased at the LD-stage. Thus, the decrease in these transcripts in our across-stage comparison could be due to increases in FSH [26,27] and IGF1 [27,29] that occur naturally in this species. Further research will be needed to identify factors that may up-regulate *cx30.9 *and *cx44.9 *gene expression during early oogenesis.

## Conclusion

In this study, we show that at least four different *cx *genes, designated *cx30.9*, *cx34.3*, *cx43.2*, and *cx44.9*, are expressed in the coho salmon ovarian follicle. Transcripts for *cx30.9 *and *cx44.9 *were highly expressed in PN-stage follicles and localized to the follicle cells and oocytes. Transcripts for *cx34.3 *were highly expressed at the mid VIT-stage and were localized only to granulosa cells. Transcripts for *cx43.2 *were highly expressed at the MAT-stage in theca and granulosa cells, and oocytes. Thus, the results of spatiotemporal analyses revealed that *cx30.9*, *cx34.3*, *cx43.2*, and *cx44.9 *were under ovarian stage and cell-type specific control during oogenesis. Further, FSH, LH, and IGF1 differentially regulated these ovarian *cx *gene transcripts *in vitro*. To our knowledge, this is the first report of IGF1 regulation of ovarian *cx *gene expression. These data indicate that the differences in spatiotemporal expression profiles and hormone-mediated regulation of these four *cx *transcripts may be related to the function of ovarian GJs during different stages of ovarian cellular differentiation. Future studies on the cell-cell communication permitted by GJs formed by these Cx proteins will be important to understanding development of the ovarian follicle in teleosts.

## Competing interests

The authors declare that they have no competing interests.

## Authors' contributions

YY participated in the study design, execution of experiments, data analysis and interpretation, and drafted the manuscript. MAM participated in the execution of experiments, performed the E2 assay, and participated in data analyses. JAL participated in study design, execution of experiments, and interpretation of data. PS acquired funding for the work, participated in study design, and assisted with data analysis and interpretation. All the authors read, edited, and approved the final manuscript.
